# A Putative Human Pheromone, Androstadienone, Increases Cooperation between Men

**DOI:** 10.1371/journal.pone.0062499

**Published:** 2013-05-22

**Authors:** Paavo Huoviala, Markus J. Rantala

**Affiliations:** 1 Department of Behavioural Sciences and Philosophy, Psychology, University of Turku, Turku, Finland; 2 Department of Biology, Section of Ecology, University of Turku, Turku, Finland; French National Centre for Scientific Research, France

## Abstract

Androstadienone, a component of male sweat, has been suggested to function as a human pheromone, an airborne chemical signal causing specific responses in conspecifics. In earlier studies androstadienone has been reported to increase attraction, affect subjects' mood, cortisol levels and activate brain areas linked to social cognition, among other effects. However, the existing psychological evidence is still relatively scarce, especially regarding androstadienone's effects on male behaviour. The purpose of this study was to look for possible behavioural effects in male subjects by combining two previously distinct branches of research: human pheromone research and behavioural game theory of experimental economics. Forty male subjects participated in a mixed-model, double-blind, placebo-controlled experiment. The participants were exposed to either androstadienone or a control stimulus, and participated in ultimatum and dictator games, decision making tasks commonly used to measure cooperation and generosity quantitatively. Furthermore, we measured participants' salivary cortisol and testosterone levels during the experiment. Salivary testosterone levels were found to positively correlate with cooperative behaviour. After controlling for the effects of participants' baseline testosterone levels, androstadienone was found to increase cooperative behaviour in the decision making tasks. To our knowledge, this is the first study to show that androstadienone directly affects behaviour in human males.

## Introduction

Pheromones are known to influence behavior in numerous animal species, but it has for long been thought that they are not important for human behavior and social interaction. However, in recent years, research on human pheromones has revealed various interesting psychological and physiological phenomena. Perhaps the most widely studied of the putative human pheromones is the compound androstadienone (4, 16-androstadien-3-one), found in relatively large quantities in male axillary sweat [1], [2]. Androstadienone has been reported to modulate women's attributions of male attractiveness [3], have, in some cases sex and context dependent, effects on mood (for example, [4], [5]), and direct attention towards emotional information [6]. Furthermore, androstadienone has been shown to maintain increased levels of salivary cortisol in women [7], activate hypothalamus in a sex [8] and sexual orientation dependent manner [9], [10], and activate brain areas related to social cognition and attention [11]. However, psychological evidence outside the, often context dependent, mood enhancing qualities remains scarce. This is especially true for male responses to androstadienone.

Importantly, the reported effects of androstadienone do not yet enable us to understand the function of androstadienone secretion. A mere increase in mood, neural or hormonal activity in a conspecific cannot be the end purpose of androstadienone secretion. Similarly to their role in insects [12], [13], reptiles [14], and other mammals [15], [16], some human pheromones have been suggested to function as a signal of mate quality [17], [3], [18]. Thus, perhaps at least some human pheromones could function as a sexual ornament, a chemical equivalent of the peacock's tail, reflecting traits such as reproductive value, good health and status. Interestingly, this view suggests that the primary effect of androstadienone would be to make males more attractive to women, while the other effects reported, such as hormonal changes [7] and increased attention to social information [6] would only be secondary reactions to the signal. For example, it could be evolutionarily adaptive to focus more closely on the social information provided by potential mating partners of high quality – resulting also in the corresponding activation of the relevant brain areas. Arguably, most reported effects of androstadienone make more sense in this light.

In many non-human animals, males' pheromones have been found to reveal social status to other males [19], [20], [21]. In humans, dominance, measured by psychometric questionnaires, has been found to be associated with a male body odour rated more attractive by females [22], although the proximate mechanism for the correlation between dominance and the attractiveness of odour is not known. Dominant behavior on the other hand has been found to be associated with high testosterone levels (e.g. [23]). Thus, by-products of testosterone production, like androstadienone [24], are possible candidates for the signal of dominance. If androstadienone acts as such a signal in humans, one could expect that it should also have observable effects on male-male social interaction. Interestingly, relatively few studies report testing androstadienone's effects on male participants. This may at least partly stem from the difficulty of measuring androstadienone's effects on social interaction, while reliably controlling for other relevant factors. In an ecologically valid environment it is very difficult to control for other factors that possibly affect behaviour, while in the laboratory it is difficult to create social situations realistic enough. Nevertheless, a possible method to do this in a controlled laboratory setting is offered by the decision making games of behavioural economics. As the exposure to androstadienone takes place intranasally, a largely similar setup to experiments studying the effects of intranasal hormone (oxytocin) exposure on social decision making [25] can be used.

Ever since its introduction as an experimental method [26], the decision making game known as the ultimatum game (and its variant, dictator game) has become an increasingly popular research method in behavioural economics, as well as in evolutionary psychology, because it offers a relatively simple method to study human cooperation and altruistic behaviour quantitatively. Colin Camerer and Richard H. Thaler [27], [28] describe the basic form of the ultimatum game: two players, the Proposer and the Responder, bargain over a fixed amount of money, for example €10. The Proposer is asked to make a proposition regarding the sharing of the €10 with the second player. The Proposer makes an offer *x* to the Responder, leaving himself with €10-*x*. If the Responder accepts the offer, he receives € *x* and the Proposer receives €10-*x*. If the Responder rejects the offer neither player gets anything. The game often takes place completely anonymously.

According to game theory principles, any self-interested Proposer should make as small offers as possible, while the Responder should accept any non-zero offer. Nevertheless, according to the experimental data, people tend to act more fairly than predicted, and also demand fair behaviour from others: Proposers offer 40–50% of the total sum on average, while Responders reject offers of less than 20% of the total sum around 50% of the time, even if the offer -and therefore the amount lost by rejecting it- equals several weeks' wages [28].

Not all ultimatum games take place anonymously, however. The ones that do not, reveal that various characteristics of the players affect decision making. Firstly, as Proposers, people are more cooperative with attractive Responders [29], [30]. Secondly, as Proposers, people with high testosterone levels (salivary or prenatal, estimated by second to fourth digit ratio) are generally more cooperative [31], [32] (however, this is not always the case, see [33]). Thirdly, as Responders, attractive people (as measured by low fluctuating asymmetry) are more likely to reject low offers [34], and finally, as Responders, people with high testosterone levels are more likely to reject low offers [35], [36].

Methodologically, an especially important experiment for the present study was conducted by Zak, Stanton and Ahmadi in 2007 [25]. In a double-blind, between groups design, male participants were exposed to either oxytocin or a saline control via nasal inhaler, and were then (after a 60 minute loading time) asked to make decisions in single-shot ultimatum and dictator games. The participants were asked to make a decision as both the Proposer and the Responder in an ultimatum game. As Responders the participants were asked to state the minimum sum they would accept from a Proposer. This minimum acceptable offer approach has the benefit of measuring likely reactions to all possible offers, and therefore provides more information; for example, extreme offers are relatively rare, and it is therefore more difficult to get information on the Responder behaviour in such a situation [28]. As the exposure to androstadienone can take place in much the same fashion as intranasal exposure to oxytocine, we can use a more or less a similar game setup to the experiment by Zak, Stanton and Ahmadi [25].

Using a similar amount of synthetic androstadienone (30 mg) as some other recent studies [7], should by far supersede the naturally occurring levels of androstadienone in human sweat. If androstadienone levels indeed function as a chemical signal of male mate quality in a dose-dependent manner, such a quantity should signal superior status, placing even the normally socially dominant males into a subordinant position. Therefore, our participants should play similarly to a situation where they are playing with an attractive and socially dominant male; they should make larger offers, because low offers are likely to be rejected and because a conspecific with such androstadienone levels is likely to be more valuable as an ally than an enemy.

Thus, we hypothesize that:

*Hypothesis 1:* As Proposers, male participants exposed to androstadienone will behave more co-operatively (make larger offers as Proposers in the ultimatum game and in the dictator game, and accept lower offers as Responders) than males of the control group.

*Hypothesis 2:* Salivary testosterone levels will have an effect on all the decision-making tasks, correlating positively with offer size as Proposers and in the dictator game, as well as with minimum acceptable offers as Responders.

*Hypothesis 3:* Androstadienone, in this context, will increase the amount of salivary cortisol during the experiment.

If androstadienone does indeed have such effects on behaviour and physiology, it would further support androstadienone functioning as a male pheromone that signals mate quality. In other words, it would support androstadienone functioning as a signaling pheromone. On the other hand, if no such effect can be observed, it would mean that either androstadienone does not signal anything to other males, it functions in a different role than predicted, or the methods used are not applicable to measure the effects.

## Methods

### Participants

A total of 40 male subjects participated in the study, of whom 20 received the androstadienone treatment, and 20 received the control treatment. The mean age of the participants was 26.03 years (SD = 4.80) and the groups did not differ significantly in this regard (t(33) = −1,096, p = ns). All of the participants signed an informed consent form. The participants were recruited through university mailing lists, message boards and personal communication, and all participants received a small monetary compensation for participating. The exact sum varied according to the participants' decisions in the decision making games. Exclusion criteria included dysosmia, a history of nasal trauma or brain injury. Furthermore, non-heterosexual participants were excluded from the analysis, because earlier research suggests that pheromones may function in a sexual orientation-dependent manner [9], [10].

### Ethics Statement

The Board of Ethics and Qualifications of University of Turku approved the experimental design of the study.

### Compounds

Thirty (30) milligrams of 4,16-androstadien-3-one, obtained from Steraloids incorporated (Newport, RI), was used for the experimental condition. The compound was then, in a crystal form, mixed with 30 milligrams of dry yeast in order to increase perceived odor similarity with the control stimulus. Sixty (60) milligrams of dry yeast was used as the control stimulus. Both stimuli were placed in opaque jars, similarly to earlier experiments [7], [37], and were stored at room temperature and kept protected from light.

### Experimental procedure

A mixed model design was used for the experiment. The experiment was conducted double-blind, and each subject participated in the study only once. All participants were tested by the same (male) experimenter (PH) and the sessions took place in an air-conditioned, temperature controlled laboratory room, between 12.00 and 18.00, each session lasting ∼60–70 minutes.

After signing the consent forms the participants were given instructions for the experiment, both orally and in writing. In addition, the participants were left with the written instructions and were encouraged to use them during the experiment. When it had been made certain that the participant had understood the instructions sufficiently, the experimenter left the room for the rest of the duration of the experiment. During this time the participants were monitored by recording the experiment via a video camera.

After the experimenter had left the room, the participants began by watching a twenty minute long relaxing aquatic video, similarly to the study by Wyart et al. [7]. The rationale was to let the participants habituate into the situation, and to buffer the baseline salivary hormonal measurements against pre-test hormonal fluctuations. According to Piferi, Kline, Younger and Lawler [38], watching a non-arousing video is the most efficient way to make participants relaxed for physiological baseline measurements.

After watching the video participants answered a twenty item questionnaire, PANAS (Positive And Negative Affect Schedule) [39], included to screen for participants who, for one reason or another, were not relaxed after watching the video.

Answering the PANAS questionnaire was followed by providing the baseline saliva sample into a 10 ml test tube. The participants were instructed to chew on a piece of Parafilm® in order to increase saliva secretion. The sample was then placed into a Styrofoam box filled with ice to keep the sample cool for the duration of the experiment.

After giving the baseline saliva sample, the participants were exposed to either androstadienone or the control stimulus. The participants opened the jar holding the stimulus, held it under their noses and took 20, five second long sniffs, with alternating nostrils, and 10 second breaks between each sniff. A computer program provided the instructions for the timing of the sniffs.

This was followed by the decision-making tasks: the ultimatum game and the dictator game. All in all, the participants had to answer three questions regarding the sharing of a total of €20.0 with another anonymous player.

In the first question, the participants acted as the *Proposer* of the ultimatum game, having to make a proposition regarding the sharing of €10.0 with the anonymous *Responder*. The rules of the games were explained, so the participants knew that low offers had the possibility of being rejected. In the second question, the participants acted as the *Responder*, having to decide the *minimum acceptable offer* they themselves would accept from another player. In the third question, the participants acted as the sole decision maker in a unilateral dictator game, and thus made a decision regarding the sharing of another €10.0, knowing that this time the other player would not get to accept or reject the offer.

Thus, a common single shot version of the games was used, (e.g. similarly to [25], [33]). This particular variation of the games was employed in order to obtain data regarding the participants' decisions in both roles of the ultimatum game; as the *Proposer* and the *Responder*. The third question, ultimatum game, was included in order to see whether the participants' decisions would change when they did not have to take another player's decisions into account. In addition to answering the questions, the participants had to place the provided coins (9×€1 and 10×10 cents, for the *Proposer decision* and the same amount for the *Dictator decision*) into envelopes, according to the decisions they had made. In the briefing before the experiment, the participants had been told that at the end of the session they would receive the money according to the actual results of the games.

The decision-making games were followed by personality questionnaires intended to function as sham tasks, and to keep the participants busy until the second (20 minutes post-exposure) saliva samples could be obtained. After these questionnaires, the participants proceeded to fill in a demographic information form, including questions on the participants' age, ethnicity and relationship status. In the form, the participants were also asked to rate the perceived *intensity* and *pleasantness* of the olfactory stimulus with a 1–10 scale. In addition, an open ended question asking them to describe and - if possible- name the stimulus was also included. Finally, a timer signaled when 20 minutes had passed from the olfactory stimulus exposure.

### Variables

#### Perceived qualities

Two variables were used to measure how the participants perceived the odor stimuli: perceived *intensity* and perceived *pleasantness*. Both were given a scale from 1 to 10, 1 meaning weak or unpleasant, respectively, while 10 meant very strong or very pleasant.

#### Psychological and behavioural variables

All in all, four variables were formed from the ultimatum and dictator game questions: *Proposer decision*, the amount of money offered to the other player in ultimatum (scale 0.0–10.0); *Responder decision*, the smallest amount of money accepted from the other player in ultimatum (scale 0.0–10.0); *Dictator decision*, the amount of money offered as the sole decision maker (scale 0.0–10.0); and a compound variable named as *Generosity*, formed by subtracting the *Responder decision* score of each participant from the *Proposer decision* score of the same participant (scale −10.0–10.0). This was done in order to see if –and to what extent- the amount of money offered to the other player differed from the offer the participants themselves were willing to accept, and whether there were between-group differences in this behaviour.

#### Hormonal variables

The saliva samples were stored in −80°C until the analysis. After thawing, and vortexing, the samples were centrifuged. The free hormone levels from the supernatant were tested with Salimetrics (State College, PA) enzyme immunoassay kits. Standard assay procedures were followed, except that only the standard and control wells were assayed in duplicate. After completing the assay, optical density of the samples was read on a Wallac plate reader (Turku, Finland) at 450 nm. The testosterone and cortisol concentrations of the samples were then interpolated by using a 4PL curve fit.

Finally, both absolute and relative change scores were calculated for the hormonal measurement results. Absolute change scores for both testosterone and cortisol were calculated for each participant as the difference of the baseline and post-exposure results, while the relative change scores were calculated by dividing the difference between the baseline and post-exposure results by the baseline value.

### Data analysis

PASW statistics version 18 was used for the data analysis.

#### Analysis of between-group differences in perceived stimulus qualities and baseline measurements

First, in order to see if the used olfactory stimuli differed in their perceived pleasantness and intensity, the *pleasantness* and *intensity* scores of the olfactory stimuli were analyzed with a t-test for independent samples. Shapiro-Wilk tests were used to test for the normality assumption of the test (p = ns, for both pleasantness and intensity), while Levene's Test of Homogeneity of Variance was used to test for the homogeneity of variance assumption (p = ns, for both scores).

Next, in order to see if the groups differed in their initial levels of testosterone and cortisol, between-group differences in baseline hormonal levels were analyzed. The testosterone samples of one participant were excluded from the analysis for extremely high values, possibly due to contamination of blood in the saliva sample. Furthermore, the cortisol samples of one participant were excluded from the analysis for very high (non-physiological) cortisol levels, possibly due to self-reported extended use of hydrocortisone cream.

The between-group differences in baseline hormonal levels were analyzed by one-way analysis of variance with *baseline cortisol* and *baseline testosterone* as dependent variables and *olfactory stimulus* (androstadienone/control) as the independent variable. Both dependent variables passed the homogeneity of variance assumption (p = ns, for both variables), but failed the assumption regarding normal distribution (Shapiro-Wilk, p<.05, for both variables). As the dependent variables were not considerably far from being normally distributed, and analysis of variance is generally considered to be relatively robust regarding violations of the normality assumption, one-way analysis of variance was used nonetheless, instead of a nonparametric test.

#### Analysis of between-group differences in decision making behavior

Next, the main effects of the olfactory stimuli used on the decision making games were tested for by running a multiple analysis of covariance (MANCOVA) with the four ultimatum and dictator scores (*Proposer decision*, the amount of money offered in ultimatum; *Responder decision*, the minimum acceptable offer stated in ultimatum; *Dictator decision*, the amount of money offered as the sole decision maker; *Generosity* the difference between participant's *Proposer decision* and *Responder decisions*) as dependent variables and the olfactory stimulus received as the independent variable. *Baseline testosterone* was entered as a covariate into the model in order to control for its effects.

None of the dependent variables passed assumption regarding the normality of distributions (Shapiro-Wilk, p<.05 for all variables). This was to be expected, however: while a majority of players favor even, close to 50/50, splits in experimental ultimatum, very few people offer more money away than what they ask for themselves, thus skewing the distributions towards non-normality.

Levene's test of equality of variances was used to test for the assumption regarding the homogeneity of variances. All p-values were non-significant so the assumption was satisfied. Observing scatterplot graphs of the relationships between the dependent variables and *baseline testosterone* revealed no interactions between the covariate and the received olfactory stimulus. Thus, MANCOVA's assumption regarding homogeneity of regression slopes was considered satisfied.

#### Analysis of between-group differences in testosterone and cortisol change

Next, the effects of the olfactory stimulus on both absolute and relative changes in hormonal levels were tested for by using one-way analysis of variance with *absolute cortisol change*, *relative cortisol change*, *absolute testosterone change* and *relative testosterone change* as dependent variables and *olfactory stimulus* as the independent variable. All but absolute change in cortisol passed the homogeneity assumption (p = .03, for absolute change in cortisol; p = ns for other variables). However, all variables failed the normality assumption (Shapiro-Wilk p<.05, for all variables). Closer examination of the distributions however resulted in the decision to use the one-way analysis of variance nevertheless.

Graphical examination of the relationships between cortisol and testosterone hinted of a possible interaction between baseline cortisol levels, the olfactory stimulus and the change in testosterone levels. A multivariate general linear model with the *absolute testosterone change* and *relative testosterone change* as dependent variables, and the *baseline cortisol* and *olfactory stimulus* as independent variables was conducted to test for the interaction.

## Results

### Between-group differences in perceived stimulus qualities and baseline measurements

Androstadienone and the control stimulus did not differ in their perceived pleasantness [*pleasantness*_Control_ M 3.65, SD 1.81; *pleasantness*_Androstadienone_ M 3.30, SD 1.53; t(38) = .660, p = .51] or intensity [*intensity*_Control_ M 4.45, SD 1.96; *intensity*_Androstadienone_ M 5.05, SD = 2.06; t(38) = −.943, p = .35]. Thus, between-group differences in behavioural or hormonal variables are unlikely to result solely from any difference in perceived odour qualities.

Interestingly, the baseline levels of testosterone correlated negatively with the perceived intensity of the control stimulus (Pearson's r = −.504, p<.05), but not for androstadienone (p = ns). The groups did not differ in their baseline levels of cortisol [*baseline cortisol*_Control_ M .249 µg/dL, SD .133; *baseline cortisol*_Androstadienone_ M .180 µg/dL, SD .083; F(1,37) = 3.65, p = .064] or testosterone [*baseline testosterone*_Control_ M 145.2 pg/mL, SD 50.03; *baseline testosterone*_Androstadienone_ M 126.6 pg/mL, SD 35.20; F(1,37) = 1.78, p = .19].

### Between-group differences in decision making behaviour

**Figure 1:**
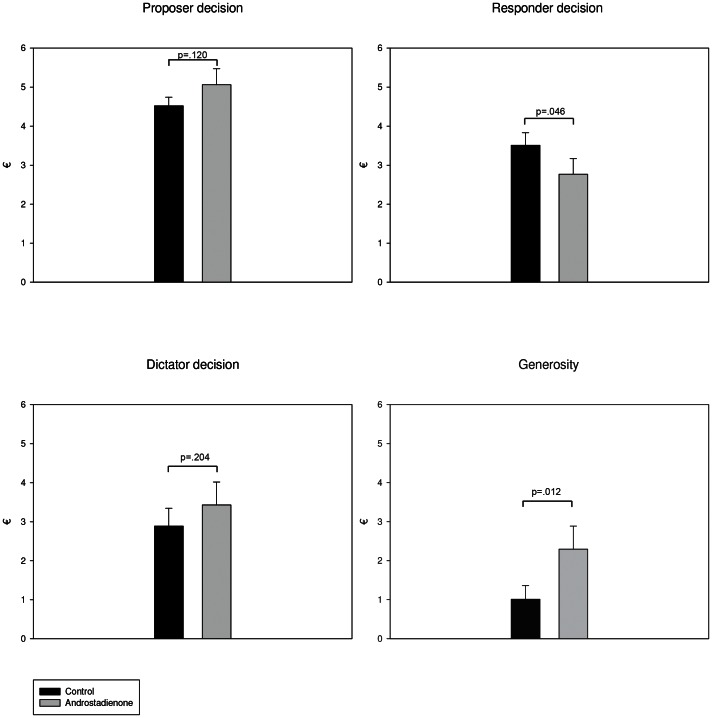
Estimated marginal means for the decision making variables. **Error bars represent standard error.**

Multiple analysis of covariance revealed that androstadienone had significant main effects on *Responder decision* [F(2,36) = 4.28, p<.05, Partial Eta^2^ .106] and *Generosity* [F(2,36) = 6.92, p<.05, Partial Eta^2^ .161], when controlling for the differences in baseline testosterone (Figure 1). The effects of the odor stimulus on the two remaining decision making tasks were non-significant, but a similar trend towards more cooperative and generous behaviour is observed nonetheless (Figure 1).

### Testosterone, cortisol and decision making behaviour

Bivariate Pearson's correlations between *baseline testosterone* and the four variables reflecting decision making behaviour revealed significant correlations between the variables (Table 1). However, no significant relationship was found for *baseline cortisol* and decision making behaviour.

**Table 1:** Pearson's correlations between decision making behaviour and baseline hormone levels.

		*Proposer decision*	*Responder decision*	*Dictator decision*
Testosterone	Pearson's r (p-value)	.267 (.10)	−.372 (<.05)	.366 (<.05)
Cortisol	Pearson's r (p-value)	.120 (.47)	−.018 (.92)	−.027 (.87)

Similarly to the observed Pearson's correlations, the baseline testosterone levels had a main effect on the behavior in all of the tasks (*Proposer decision* F(2,36) = 4.07, p = .05, Partial Eta^2^ .102; *Responder decision* F = 8.56, p<.01, Partial Eta^2^ .192; *Dictator decision* F = 6.95, p<.05, Partial Eta^2^ .162; *Generosity* F = 12.68, p<.01, Partial Eta^2^ .261).

### Between-group differences in testosterone and cortisol change

Unlike predicted, the *olfactory stimulus*, did not have a main effect on absolute or relative change scores for neither cortisol [*absolute change in cortisol* M −.061, SD .078, F(1,37) = 1.710, p = 0.13; *relative change in cortisol* M −.209, SD = .409, F(1,37) = .466, p>.05] nor testosterone [*absolute change in testosterone* M −15.85, SD 31.21, F(1,37) = .846, p = .35; *relative change in testosterone* M −.120, SD 1.61, F(1,37) = 1.54, p>.05].

Surprisingly, the amount of *baseline cortisol* and *olfactory stimulus* had an interaction effect on both, the absolute change in testosterone [*absolute change in testosterone*, F(3,34) = 5.53, p<.05, Partial Eta^2^ .140; *relative change in testosterone*, F(3,34) = 3.96, p = .055, Partial Eta^2^ .104]. In the control group, high levels of baseline cortisol resulted in an increase –or in most cases, a smaller decrease- in testosterone levels, while in the androstadienone receiving group, a high baseline level of cortisol resulted in a slightly larger decrease in testosterone levels.

## Discussion

The purpose of this study was to find out if the putative male pheromone, androstadienone, carries information relevant for social decision making, as measured by behaviour in ultimatum and dictator games. Furthermore, we wanted to find out whether androstadienone affects male salivary hormone levels (cortisol and testosterone), as seen in females [7]. Our first hypothesis was that male participants exposed to androstadienone behave more co-operatively in the ultimatum and dictator games. This hypothesis received support: when controlling for participant baseline testosterone levels, the androstadienone receiving group accepted significantly lower offers as Responders, and the difference between Proposer offers and the minimum acceptable offers was significantly higher than in the control group (meaning that participants offered more and asked for less). There was also a tendency in the androstadienone receiving group to make larger offers as Proposers and as sole decision makers in ultimatum. Thus, it seems that androstadienone increased cooperation in ultimatum and dictator.

Secondly, we hypothesized that, similarly to some earlier studies [31], [32], the participants' salivary testosterone levels would have an effect on the decision making, increasing the size of offers as Proposers and as the sole decision maker in dictator, but also increasing the *minimum acceptable offer* as the Responder. As predicted, baseline testosterone did correlate positively with larger offers, but interestingly, the correlation was negative in regard to *minimum acceptable offers*. Thus, in the present study, males with high salivary testosterone levels made larger offers but also accepted smaller offers than males with lower testosterone levels.

Thirdly, we hypothesized that androstadienone would increase male cortisol levels, similarly to women [7]. This hypothesis was not supported by our findings. However, an interaction between baseline levels of cortisol and androstadienone exposure was found, hinting that androstadienone exposure might possibly affect the change in participants' testosterone levels during the experiment.

Part of the findings were entirely novel, while part of them differed slightly from those of earlier studies. Relatively few earlier studies have shown androstadienone having any effects on human males, although it has been shown to decrease positive stimulated mood [5], modulate mood and memory while watching mood-inducing videos [39], and direct attention towards social information [6]. To our knowledge, this is the first study to show that androstadienone directly affects behaviour in human males. However, it is notable that the found effects were statistically significant only after controlling for the participants' baseline testosterone levels. This might be one important factor when looking for novel effects of androstadienone in the future. As others have earlier pointed out: the effects of androstadienone seem to depend on the context. Naturally the qualities, such as testosterone levels of the recipient, are a part of this context.

The results regarding the role of testosterone in decision making behaviour were in concordance with some of the earlier results: similarly to some of the earlier studies [31], [32] higher testosterone levels correlated positively with higher offers as a Proposer. These results differ from the ones by Zak et al. [33], but the reason may be that in that particular study the participants' testosterone levels were artificially increased. However, high testosterone males have earlier been reported to be more likely to reject low offers as Responders [35]. Nevertheless, in our study testosterone levels correlated negatively with the minimum acceptable offers. This may be due to differences in the experimental setup, cultural differences, or the fact that Burnham's [35] participants were graduate students who had actually studied microeconomics and game theory, while our sample was more varied.

Overall, our results can be interpreted as further supporting the functioning of androstadienone as a human pheromone. Moreover, they provide new information on the nature of this putative pheromone: although many of androstadienone's effects might be sex or even sexual orientation-specific [9], [10], androstadienone clearly also affects male social behavior. As our participants performed similarly to experiments where playing with attractive conspecifics [29], [39], our results can be interpreted as supporting the hypothesized role of androstadienone as a signal of male mate quality [3], [17], although more research is naturally needed on the participant before any definite conclusion can be reached.

One possible ultimate level explanation for why androstadienone –or any signal of mate quality and status- increases cooperation in males, is that in the evolutionary history of our species, cooperation, rather than aggression, may have been a more adaptive form of behaviour when interacting with same-sex conspecifics of high status and mate quality. Interestingly, if salivary testosterone is really linked to status, high status males also seem to favor cooperation more strongly. This would provide especially strong incentives for others to cooperate with high status individuals, as cooperation brings both immediate and delayed benefits, while costs of a possible conflict are likely to increase with the status of the opponent.

One question arises from this: if cooperation in resource sharing comes with such benefits, who, then, does not cooperate? Splitting the participants in two groups, according to whether individuals baseline testosterone levels are in the upper 50% or lower 50% of the sample, shows that by a clear margin, the most uncooperative males are the ones belonging to the lower 50% *and* the control group. For example, a mean offer as the sole decision maker in the dictator game is €1.68, compared to the €4.06 by the high testosterone males in the androstadienone group. This suggests an interesting phenomenon: low status individuals do not treat each other fairly, while high status individuals are fair to both low and high status individuals.

In order to better understand this behaviour, we must look more closely at the possible benefits and costs involved in cooperative behaviour regarding resource sharing. The benefits are that: apparently such behaviour is considered attractive by the opposite sex [29], and such acts may produce delayed benefits via direct reciprocation or reputation increase. On the other hand, the costs are the part of the resource lost by sharing. While the benefits are likely to be the same for individuals of both high and low testosterone levels (and according status), the costs of resource sharing arguably increase together with the scarcity of the resource. Therefore, it could be that low testosterone and low status individuals value the resource -the immediate benefits- more highly. It may, for example, be that resources are harder to come by for them. Perhaps cooperation and generosity are only a good strategy for those who can truly afford it. Those who cannot, must rely on more opportunistic tactics.

### Conclusion

Our study was the first to integrate the two distinct branches of research: human pheromone research and research on decision making behavior. As such, it produced novel findings and, arguably, opens up interesting new possibilities for future research. Variations of the study could easily be conducted by altering the olfactory stimulus used (for example, by using estratetraenol or sweat collected in t-shirts or cotton pads) and various qualities of the participants, in order to find out more about human pheromones and cooperation, both within and between sexes.

Especially important would be to delve further into the relationship between androstadienone and attractiveness, in order to see how reliably androstadienone increases attraction, and to what extent the effect depends on the concentrations of the stimulus. This could actually aid in integrating the apparently non-related findings regarding the effects of androstadienone into a more sensible theoretical framework. If more evidence is found to support the hypothesis of androstadienone as the ‘chemical equivalent of a peacock's tail’, many of its reported effects may be better understood as secondary responses to the information carried by the signal. Moreover, even if the signal is the same for both sexes, responses need not –and indeed should not- be. An attractive and dominant male can be a valuable potential mating partner for a female, but precisely for the same reason, a competitor for another male.
